# Analysis of anxiety-related factors amongst frontline dental staff during the COVID-19 pandemic in Yichang, China

**DOI:** 10.1186/s12903-020-01335-9

**Published:** 2020-11-26

**Authors:** Suli Zhao, Jing Cao, Rongcan Sun, Lin Zhang, Beibei Liu

**Affiliations:** 1 Department of Stomatology, The People’s Hospital of China Three Gorges University/ The First People’s Hospital of Yichang, 2 Jiefang Road, Yichang, 443000 China; 2 Department of Environmental Health Sciences, Yale School of Public Health, 60 College Street, New Haven, CT 06520 USA; 3 Hubei Xinqingjia Psychological Counseling Co., Ltd., 129 Yanjiang Avenue, Yichang, 443000 Hubei China

**Keywords:** COVID-19, Anxiety disorders, Becker anxiety inventory, Frontline dental staff

## Abstract

**Background:**

Dental staff were characterized with the tolerance of enduring stress and they are at a high risk to respiratory infectious disease. This study compared the anxiety level of the frontline dental staff (FDS) to the general public in Yichang during the coronavirus disease of 2019 (COVID-19) pandemic and examined potential explanatory factors to the differences.

**Methods:**

Two online questionnaires were used separately to collect data from FDS and the general public. The Chinese version of Beck Anxiety Inventory (BAI) was included for the assessment of anxiety. Firstly, a Chi-square test was conducted to compare the anxiety state between these two groups. Then, a bivariate analysis using Cramer’s V and Eta squared was conducted to find the potential factors. Lastly, a binary logistic regression was performed to examine the association between potential factors and the anxiety state of FDS.

**Results:**

In general, FDS were 4.342 (95% CI: 2.427–7.768) times more likely to suffer from anxiety disorders than the general public. The bivariate analysis showed that age, Level Three Protective Measures (PM-3), conflicts with patients and/or colleagues were moderately associated with the anxiety state of FDS. But the knowledge of COVID-19, the treatment to suspected or confirmed cases both had a weak association with the anxiety among FDS. Conversely, workload, the exposure to potential infectious substance and conducting aerosol generated performance were not significantly related to the anxiety of FDS. As the model indicated, an elder age and PM-3 protective measures could lower the anxiety state of FDS, whereas the conflict with patients or/and colleagues would worsen it.

**Conclusions:**

During the COVID-19 pandemic, FDS were more likely to suffer from anxiety disorders than the general public. An elder age, sufficient personal protective measures and good relationships with colleagues and patients would help them to maintain good mental health.

## Background

Since the first confirmed case was diagnosed in December 2019 in Wuhan, China, coronavirus disease of 2019 (COVID-19) has exerted adverse impact on every aspect of daily life [1]. This disease is caused by severe acute respiratory syndrome coronavirus 2 (SARS-CoV-2), and its transmission can occur after the close contact with infected individuals via their body fluids and the respiratory droplets and aerosols they produced through coughing, sneezing, talking and other activities [2].

As was known, dental treatments may involve the use of dental hand pieces, ultrasonic scalers, water-air syringes, etc., which normally generate aerosols, sprays, spits and splashes of saliva, blood or other transport media of SARS-CoV-2 [3–5]. Therefore, dental settings in China were closed for a period of time according to the lock-down policy in different areas, during which only urgent patients were treated and most cases had been postponed. After the spreading of SARS-CoV-2 had been curbed, each area gradually resumed dental services, and precautions (such as the sanitization of equipment and operatories, the use of the personal protective equipment (PPE) and the management of medical waste) have been enforced in accordance to the guideline issued by National Health Commission of the People’s Republic of China [6]. Before returning to work, all dental staff are required to be trained with the basic knowledge of COVID-19. Moreover, some dental settings have followed the Level Three Protective Measures (PM-3) (including medical uniform, gown, medical cap, N95 respirator, goggles, face shield, medical hazmat suit, gloves, and medical shoe covers [7]) to minimize the harm to FDS which includes dental interns, dental nurses and general dentists.

These measures of protection and disinfection could effectively prevent FDS from contracting the disease, however, their mental health also needs concern [8, 9]. Studies showed that, during the spreading of SARS-CoV-2 in China, frontline medical staff were more likely to suffer from anxiety and depression disorders than the general public [10, 11]. One of these studies also showed that age was associated with the mental state of the general public [12]. Although the general situation in China was under control before the resumption of dental settings, the risk of infection still exists, for outbreaks and asymptomatic cases were reported occasionally, which could increase fear and anxiety among FDS. As for precautious approaches, it was reported that constant wearing of PPE could cause discomfort, such as breathlessness and skin injury [7]. In contrast, the knowledge of COVID-19 pandemic might ease the anxiety of FDS as it did to the Korean college students in another study [13].

Even regardless of the influence posed by COVID-19, dental staff were characterized as susceptible to enduring stress [14]. Studies had investigated this question and relevant stress factors were found, among which patient relations [14], dental procedure-related factors [14] and heavy workload [15] had been reported. These factors are still likely to occur during the COVID-19 pandemic.

Therefore, this study investigated the anxiety state of the FDS after the resumption of dental settings in Yichang, a province administrated city with a population of approximately 4 million and located 300 km away from Wuhan. Based on previous research and the spread of COVID-19, there were two aims of this study: i) to compare the anxiety state of FDS to that of the general public after the resumption of dental services. ii) investigate the potential factors (age, the exposure to potential infectious substance from patients, the treatment to suspicious or confirmed cases, aerosol generated performance, PM-3 protective measures, the knowledge of COVID-19, workload, the conflict with patients and/or colleagues) associated with the anxiety state of FDS after the resumption.

## Methods

### Participants and data collection

Two online questionnaires were used separately for collecting data from the general public (Additional file 3, Additional file 4) and the FDS (Additional file 1, Additional file 2) in Yichang during a period of time from the 2nd to the 13th of May 2020. The questionnaire for general public (Additional file 3, Additional file 4) only contained questions of personal information and the Beck Anxiety Inventory (BAI) which was used to assess their anxiety state. As for FDS (Additional file 1, Additional file 2), besides these two parts, a short test on their knowledge of COVID-19 were included, and more questions relevant to their work environment and protective measures had been asked. These questionnaires were distributed through social media, such as WeChat and QQ, and were accessible via phone, computer and tablet. Each participant had been informed of this study and their consent had been asked.

In general, 330 questionnaires were distributed to the FDS in Yichang and 280 were received with a completion rate of 84.85%. Meanwhile, 330 questionnaires were distributed to the general public in this area and 285 were received with a completion rate of 86.36%. As for data set provided by FDS, since FDS only referred to dental interns, dental nurses and general dentists, data from 11 volunteers were excluded. Therefore, the sample size of FDS is 269. As for the general public, in order to compare with FDS, only participants of 20–56 were included, leading to a sample size of 258 for the general public group.

### Assessment of anxiety state of all participants

The Chinese version of BAI [16], which is reliable and valid [17, 18], was used in this study to assess the anxiety state of both groups. BAI is a self-rated inventory with 21 items measuring cognitive, emotional and physical aspects of anxiety disorders. Each item was provided with four answers of different scores (1 = not at all, 2 = mildly, 3 = moderately, 4 = severely). The summary of the score of 21 items were applied with the formula Y = int (1.19*x) to calculate the final score for each participant [18]. A final score equals to or was higher than 45 would be counted as having anxiety disorders or otherwise [19]. Hence, the outcome variable is binary.

### Potential factors associated with the anxiety state of FDS

Other questions relevant to potential factors were included in the questionnaire for FDS. They were as follow: i) the number of working days per week, ii) the number of working hours per day, iii) the number of working hours between breaks, iv) whether they often performed aerosolization procedures, v) whether they had conflicts with colleagues and/or patients in the last 6 months, vi) whether they had performed treatment on confirmed or suspected cases of COVID-19, vii) whether their skin or wounds were exposed to the blood, saliva, or other body fluids of patients, viii) whether the guideline of PM-3 had been followed in their office.

Another potential factor investigated was the knowledge of COVID-19 among FDS, six multiple choice questions of COVID-19 were used to conduct the assessment. The answers to each question was set according to the Diagnosis and Treatment Protocol for Novel Coronavirus Pneumonia (Trial Version 7) [20]. These questions covered the following aspects regarding COVID-19: i) the common symptoms, ii) the incubation period, iii) the main routes of transmission, iv) the effective measures and chemicals for cleaning and disinfection, v) the discharging criteria for confirmed cases, vi) the susceptibility of people to this disease. There was only one correct answer among four choices for each question. A correct answer was counted as 1 point, otherwise 0 point. The summary score was calculated to assess the knowledge of COVID-19 among FDS.

### Statistical analysis

In this study, the statistical analysis of data was performed via Microsoft Excel version 16.43 (Microsoft Corporation, Redmond, Washington, USA) and SPSS version 26.0 (International Business Machines Corporation, Armonk, New York, USA). Firstly, it was a between group design for the examination of the anxiety state between FDS and the general public. Therefore, after the final scores for participants were calculated, they were classified into two groups as ‘have anxiety disorders’ or ‘doesn’t have anxiety disorders’. Then a Chi-square Test was conducted to determine the difference between FDS and the general public on their anxiety state.

Secondly, the potential factors relating to the anxiety state of the FDS was investigated. Since the relevant questions yielded categorical and numerical data, a Chi-square test and the Cramer’s V were calculated when the association between two categorical variables were studied, and the calculation of Eta squared was applied to study the association between a categorical variable and a numerical variable. The association between each potential factor and the anxiety state of FDS was assessed, so as to the relations between every two potential factors, in order to minimize the influence brought by highly correlated potential factors and to find the potential factors that were moderately or strongly associated with the anxiety state of FDS.

Finally, a binary logistic regression model was developed to examine the relationships between the predictor variables (potential factors) and binary outcome variable (The anxiety state of the FDS).

## Results

### Descriptive results

The descriptive analysis was as follow. The features for gender and age of FDS and the general public were presented in Table 1. The average age of FDS was approximately 13 years younger than that of the general public group.

**Table 1:** The gender and age distributions among FDS and the general public

Variable	FDS	General public
In total(n)	269	258
Gender (Percentage)		
Male	85 (31.60%)	100(38.76%)
Female	184 (68.40%)	158 (61.24%)
Age		
Range	20–56	23–56
Average (SD)	27.20 (8.40)	40.53 (10.00)

As for some potential factors, a relatively heavy workload was perceived among FDS. In Table 2, the majority of FDS worked no more than 5 days per week, whereas 28.25% of them worked longer. There were 11.89% of the FDS worked more than 8 h a day and 29.00% of the FDS continued working more than 2 h before a break.

**Table 2:** Descriptive results of workload among FDS

Variable	Frequency (%)
Total	269(100.00%)
Working days per week	
0–4 days	7(2.60%)
5 days	186(69.15%)
6–7 days	76(28.25%)
Working hours per day	
< 4 h	35(13.01%)
[4, 6) hours	49(18.22%)
[6, 8) hours	153(56.88%)
≥ 8 h	32(11.89%)
Working hours between breaks^a^	
< 1 h	68(25.28%)
[1, 2) hours	123(45.72%)
[2, 4) hours	59(21.93%)
≥ 4 h	19(7.07%)

^a^Time intervals less than 5 min were not counted as a break

Then, the average score of FDS on the COVID-19 knowledge was 2.46 (SD = 1.33). The percentage of the correct answer for each question was shown in Table 3. FDS were more likely to choose wrong answer on effective sanitary chemicals, the main routes for transmission and the discharging criteria.

**Table 3:** Descriptive results of knowledge of COVID-19 among FDS

Questions	Frequency (%)
Total	269(100.00%)
The common symptoms	
Correct	221(82.16%)
Incorrect	48(17.84%)
The incubation period	
Correct	158(58.74%)
Incorrect	111(41.26%)
The main routes of transmission	
Correct	91(33.83%)
Incorrect	178(66.17%)
Cleaning and disinfection^a^	
Correct	68(25.28%)
Incorrect	201(74.72%)
The discharging criteria	
Correct	41(15.24%)
Incorrect	228(84.76%)
The susceptibility of people^b^	
Correct	83(30.86%)
Incorrect	186(69.14%)

^a^The effective measures and chemicals for cleaning and disinfection

^b^The susceptibility of people to COVID-19

### Comparison of anxiety state between FDS and the general public

As the result showed, 60 out of 269 FDS (22.30%) suffered from anxiety disorders and 16 out of 258 ordinary people (6.20%) suffered from anxiety disorders. There was a significant association (χ^2^ = 27.671, *p* < 0.01) between being FDS and having anxiety disorders. Based on the odds ratio, the likelihood for FDS to suffer from anxiety disorder was 4.342 (95% CI: 2.427–7.768) times higher than that of the general public.

### Bivariate analysis

As for bivariate analysis, firstly, the associations of several potential factors (categorical variables) with the anxiety state of FDS were assessed. As was shown in Table 4, factors that were significantly associated with the outcome variable (the anxiety state of FDS) included social relationships with colleagues and patients, the treatment to suspected or confirmed cases, and adopting PM-3. But the treatment to suspected or confirmed cases only had a weak association with the outcome variable, since the Cramer’s V was 0.139.

**Table 4:** Associations between potential factors and anxiety disorders of FDS (categorical variables)

Variable	Anxiety state		χ^**2**^	Cramer’s V	***P-*** value
	Yes	No			
	(***n*** = 60)	(***n*** = 220)			
Gender			2.441	0.095	0.118
Male	14	71			
Female	46	138			
Aerosolization procedures^a^			0.009	0.006	0.926
Yes	44	152			
No	16	57			
Conflicts with colleagues and/or patients^b^			13.833	0.227	< 0.001
Yes	14	14			
No	46	195			
Treatment on suspected or confirmed cases^c^			5.200	0.139	0.023
Yes	11	17			
No	49	192			
Exposure to potential infectious substance ^d^			3.252	0.110	0.071
Yes	18	40			
No	42	169			
PM-3^e^			10.246	0.195	0.001
Yes	43	185			
No	17	24			

^a^Did you often perform aerosolization procedures?

^b^Did you have conflicts with colleagues and/or patients in the last 6 months?

^c^Have you performed treatment on suspected or confirmed cases of COVID-19?

^d^Have your skin or wounds been exposed to the blood, saliva, or other body fluids of patients?

^e^Does your workplace follow PM-3 guideline?

In addition, other factors (numerical variables) were also listed as follow. Table 5 showed the Eta squared of each predictor variable. It was shown that there were four factors (a. the number of working days per week, b. the number of working hours per day, c. their knowledge of COVID-19, d. the number of trainings organized by workplace) that had no or weak association with the anxiety state of FDS. But the age factor had a moderate association.

**Table 5:** Associations between potential factors and anxiety disorders of FDS (numerical variables)

Variable	η^**2**^
Age	0.199
The number of working days per week	0.042
The number of everyday working hours per day	0.028
The number of hours between breaks	0.014
Knowledge of COVID-19	0.128

### Binary logistic regression

A binary logistic regression model was then developed to see the relations between the predictor variables and outcome variable (the anxiety state of FDS). The Chi-square for this model was 46.473 (*p* < 0.001), suggesting that the model fitted the data well. The overall classification accuracy rate of the model was 81.78%. Specifically, 95.69% of the FDS who have no anxiety disorders were accurately classified, but the accuracy rate was lower for FDS who had anxiety disorders (33.33%). The coefficients of all predictor variables were presented in Table 6. These three predictor variables were significantly related to the anxiety state of FDS and predictive to the outcome variable. With the increase of age, FDS were less likely to suffer from anxiety disorders. Similarly, applying PM-3 at dental settings would predict the reduction of FDS with anxiety disorders. On the contrary, FDS who had conflicts with colleagues and/or patients were more likely to develop anxiety disorders. More specifically, based on the odds ratio, FDS with an elder age were less susceptible to anxiety disorders by a factor of 0.857. The odds of suffering from anxiety disorders was 0.243 times lower for FDS with PM-3 than that of FDS without PM-3. Lastly, FDS who had conflicts with colleagues and/or patients were 2.991 times more likely to suffer from anxiety disorder than who did not.

**Table 6:** Results of binary logistic regression analysis

Variable	Coefficient	OR (95%IC)	***P***-value
Age	−0.154	0.857 (0.794–0.925)	< 0.001
PM-3^a^	−1.415	0.243 (0.105–0.561)	0.001
Conflicts with colleagues and/or patients^b^	1.095	2.991 (1.238–7.227)	0.015
Constant	3.609	36.7929	0.001

^a^Does your workplace follow PM-3?

^b^Did you have conflicts with colleagues and/or patients in the last 6 months?

## Discussion

The comparison of the anxiety state between FDS and the general public showed that during the COVID-19 pandemic, FDS were more susceptible to anxiety disorders than the general public, which was similar to the study of medical staff during the COVID-19 outbreak [9–11].

FDS with an elder age were less likely to develop anxiety disorders than younger ones, which had also been observed among the public in the outbreak of COVID-19 [12]. Also, personal protective measures (PM-3) could ease the anxiety of FDS, potentially because this measure may lower the fear caused by COVID-19. Lastly, it was the same as before, during the COVID-19 pandemic, that having less conflicts and good communications with colleagues and patients could help FDS experience less anxiety [14], since the fear of being judged negatively or embarrassed could result in social anxiety disorders [20].

Different from previous research, this study suggested that workload did not significantly influence the anxiety state of FDS. This could be related to their conscientiousness personality trait which would assist people in finding positive solutions to difficulties [21] and lowering anxiety [22, 23]. Although performing aerosol procedures [3, 4, 15], providing treatment to suspected or confirmed cases and the exposure to the body fluids of patients were perceived to be risky [2, 5], they did not closely related to the anxiety state of FDS, which could be the result of sufficient protective measures. Although better knowledge of social disease related to less anxiety among Korean college students [13] and positive attitude among Chinese residents [24], the knowledge of COVID-19 only had a weak association to the anxiety state of the FDS. This may result from the variation of symptoms among infected individuals and the occasional updates of COVID-19 guideline.

Therefore, more attention needs to be paid to the younger staff. Approaches, such as sufficient personal protective measures and providing support from colleagues, would help FDS stay away from anxiety disorders. Also, considering the features of COVID-19, regular trainings of necessary information were still in need.

There were several limitations indicated. Firstly, sample selection bias may occur in this study. Because online questionnaires were used to avoid the contact with people, participants in this study were mostly voluntary internet users. In order to minimize this disadvantage, data had been collected from different areas in Yichang and a large sample size was used. Secondly, the average age of FDS is much younger than that of the general public. This is because many FDS are in their 20s or 30s. Their physical health condition is more suitable for intense working pace than elder FDS. Considering the age among FDS ranges from 20 to 56, and in order to mitigate the influence of different age groups, participants younger than 20 or elder than 56 had been excluded from the general public group, leading to a range from 23 to 56 years old. Also, age had been studied as a potential factor. Thirdly, only a limited number of factors were investigated in this study and this model predicted 33.33% of FDS who suffered from anxiety disorders, future research could look into more anxiety-related factors to further our understanding.

## Conclusions

This study found that, during the COVID-19 pandemic, FDS were more likely to suffer from anxiety disorders than the general public after returning to work at dental settings. In addition, we found that the anxiety state of FDS was significantly associated with age, the implementation of PM-3, conflicts with colleagues and/or patients and the knowledge of COVID-19. More specifically, age and PM-3 were adversely associated with the anxiety state of FDS, whereas conflicts with colleagues and patients were positively related to the anxiety state of them. Interestingly, workload, the exposure to potential infectious substance and conducting aerosol generated performance were insignificant factors to the perceived anxiety disorders among FDS. In addition, the knowledge of COVID-19 and the treatment to suspected or confirmed cases were weakly associated with the anxiety state of FDS. Hence, according to this study, younger FDS requires more attention during the resumption of dental settings. Also, some practical approaches could be adopted, such as sufficient personal protective measures and the support provided by colleagues. Although the knowledge of COVID-19 had a weak association with the anxiety state of FDS, regular trainings regarding important information of COVID-19 are still necessary.

## Supplementary Information

**Additional file 1**.

**Additional file 2**.

**Additional file 3**.

**Additional file 4**.

## Data Availability

The data generated and analyzed during this study are available from corresponding author on reasonable request. The data are not publicly available due to privacy restrictions.

## Abbreviations

COVID-19: Coronavirus Disease 2019
FDS: Frontline Dental Staff
BAI: Beck Anxiety Inventory
PM-3: Level Three Protective Measures
PPE: Personal Protective Equipment
