# Supplementary material for: Analysis of anxiety-related factors amongst frontline dental staff during the COVID-19 pandemic in Yichang, China
Source: BMC Oral Health. 2020 Nov 26;20:342. doi: 10.1186/s12903-020-01335-9 (PMC7689639; doi:10.1186/s12903-020-01335-9)
Supplement: Supplementary file 1 — Additional file 1. [file 12903_2020_1335_MOESM1_ESM.docx]

**The questionnaire of the recent work condition and anxiety state among frontline dental staff in Yichang during the COVID-19 pandemic**

Recently, everything is gradually back to normal across the country but the risk of COVID-19 still exists. Dental staff are regarded as a group of people with heavy workloads and are at risk of cross-infection. Moreover, during the COVID-19 pandemic, frontline dental staff are working under great pressure. In this study, we want to learn about: 1. the anxiety state of frontline dental staff in Yichang during the COVID-19 pandemic and 2. the potential factors to the anxiety state of frontline dental staff in Yichang during the COVID-19 pandemic. Hopefully, this study can assist dental institutions to provide better protective measures and mental support to frontline dental staff.

This questionnaire contains some questions asking about your personal information and your anxiety state, if you have any uncomfortable feeling, you can stop the survey at any time you like. Your data will be collected anonymously and will be stored on password-protected computers.

If you have any questions, please feel free to contact:

- Beibei Liu, Email: lpxblnottingham@qq.com
- Suli Zhao, Emial: zhaosuli-9@163.com
- Rongcan Sun, Email: rongcan.sun@yale.edu

To participate in this survey, you need to be a frontline dental staff who is working in Yichang.

After knowing the information above, are you willing to take part in this survey?

1. Yes
2. No

**Thank you for your precious time, please follow the instructions to complete the questions, thank you.**

1. Gender
2. Male
3. Female
4. Age (completion)
5. Occupation (completion)

**Please answer the following questions according to your work conditions, thank you.**

1. Recently, how many days do you work every week recently? (completion)
2. Recently, how many hours do you work every day recently? (completion)
3. Recently, how many hours do you work continuously before you have a break (less than 5 minutes is not counted as a break)? (completion)
4. Do you often perform aerosolization procedures?
5. Yes
6. No
7. Have you had conflicts with colleagues and/or patients in the last six months?
8. Yes
9. No
10. Have you performed treatment on confirmed or suspected cases of COVID-19?
11. Yes
12. No
13. Have your skin or wounds been exposed to the blood, saliva, or other body fluids of patients?
14. Yes
15. No
16. Does your office follow the guideline of PM-3?
17. Yes
18. No

**Please answer the following questions according to what you know about the COVID-19, thank you.**

1. Which option is the main symptoms of COVID-19?（answer is A）
2. having a fever, dry cough, lacking in strength
3. having a stuffy nose, sore throat, lacking in strength
4. having a fever, sore throat, diarrhea
5. having a fever, having a stuffy nose, sore throat
6. Which option is the incubation period of COVID-19? (answer is B)
7. 3-7 days
8. 1-14 days
9. 1-28 days
10. more than 28 days
11. Which option is the main routes of transmission of COVID-19? (answer is A)
12. Droplet, touch, respiratory aerosol in a confined space
13. droplet, touch, excrement
14. touch, respiratory aerosol in a confined space, excrement
15. droplet, touch, respiratory aerosol in a confined space, excrement
16. Which of the following option is not the effective measures and chemicals for cleaning and disinfection for COVID-19? (answer is D)
17. ether, 75% ethanol, chlorine disinfectant
18. 75% ethanol, chlorine disinfectant, being kept in a temperature which is no less than 56 Celsius for 30 minutes
19. 75% ethanol, chlorine disinfectant, peracetic acid
20. 75% ethanol, peracetic acid, chlorhexidine
21. Which of the following option is the discharging criteria for confirmed COVID-19 cases? (answer is C)
22. body temperature has been normal for more than 3 days; there’s obvious improvement in respiratory symptoms
23. there’s improvement in respiratory symptoms; body temperature is normal; showing negative in two nucleic acid amplification tests
24. body temperature has been normal for more than 3 days; there’s obvious improvement in respiratory symptoms; CT scan shows that the inflammation in the lungs has improved; showing negative in two nucleic acid amplification tests (The second test is 24 hours or more than 24 hours later than the first test)
25. body temperature has been normal for more than 7 days; there’s obvious improvement in respiratory symptoms; CT scan shows that the inflammation in the lungs has improved; showing negative in two nucleic acid amplification tests (The second test is 24 hours or more than 24 hours later than the first test)
26. Which group of people is more susceptible to COVID-19? (answer is D)
27. children and old people
28. old people
29. young people and old people
30. all age groups

**Below is a list of common symptoms of anxiety. Please carefully read each item in the list. Indicate how much you have been bothered by that symptom within the last 7 days, including today. Choose the most suitable description.**

1. numbness or tingling
2. not at all
3. mildly, but it didn’t bother me much
4. moderately-it wasn’t pleasant, but I can tolerate.
5. severely-I can barely tolerate it.
6. feeling hot
7. not at all
8. mildly, but it didn’t bother me much
9. moderately-it wasn’t pleasant, but I can tolerate.
10. severely-I can barely tolerate it.
11. wobbliness in legs
12. not at all
13. mildly, but it didn’t bother me much
14. moderately-it wasn’t pleasant, but I can tolerate.
15. severely-I can barely tolerate it.
16. unable to relax
17. not at all
18. mildly, but it didn’t bother me much
19. moderately-it wasn’t pleasant, but I can tolerate.
20. severely-I can barely tolerate it.
21. fear of worst happening
22. not at all
23. mildly, but it didn’t bother me much
24. moderately-it wasn’t pleasant, but I can tolerate.
25. severely-I can barely tolerate it.
26. dizzy or lightheaded
27. not at all
28. mildly, but it didn’t bother me much
29. moderately-it wasn’t pleasant, but I can tolerate.
30. severely-I can barely tolerate it.
31. heart pounding/racing
32. not at all
33. mildly, but it didn’t bother me much
34. moderately-it wasn’t pleasant, but I can tolerate.
35. severely-I can barely tolerate it.
36. unsteady
37. not at all
38. mildly, but it didn’t bother me much
39. moderately-it wasn’t pleasant, but I can tolerate.
40. severely-I can barely tolerate it.
41. terrified or afraid
42. not at all
43. mildly, but it didn’t bother me much
44. moderately-it wasn’t pleasant, but I can tolerate.
45. severely-I can barely tolerate it.
46. nervous
47. not at all
48. mildly, but it didn’t bother me much
49. moderately-it wasn’t pleasant, but I can tolerate.
50. severely-I can barely tolerate it.
51. feeling of chocking
52. not at all
53. mildly, but it didn’t bother me much
54. moderately-it wasn’t pleasant, but I can tolerate.
55. severely-I can barely tolerate it.
56. hands trembling
57. not at all
58. mildly, but it didn’t bother me much
59. moderately-it wasn’t pleasant, but I can tolerate.
60. severely-I can barely tolerate it.
61. shaking/unsteady
62. not at all
63. mildly, but it didn’t bother me much
64. moderately-it wasn’t pleasant, but I can tolerate.
65. severely-I can barely tolerate it.
66. fear of losing control
67. not at all
68. mildly, but it didn’t bother me much
69. moderately-it wasn’t pleasant, but I can tolerate.
70. severely-I can barely tolerate it.
71. difficulty in breathing
72. not at all
73. mildly, but it didn’t bother me much
74. moderately-it wasn’t pleasant, but I can tolerate.
75. severely-I can barely tolerate it.
76. fear of dying
77. not at all
78. mildly, but it didn’t bother me much
79. moderately-it wasn’t pleasant, but I can tolerate.
80. severely-I can barely tolerate it.
81. scared
82. not at all
83. mildly, but it didn’t bother me much
84. moderately-it wasn’t pleasant, but I can tolerate.
85. severely-I can barely tolerate it.
86. indigestion
87. not at all
88. mildly, but it didn’t bother me much
89. moderately-it wasn’t pleasant, but I can tolerate.
90. severely-I can barely tolerate it.
91. fainted/lightheaded
92. not at all
93. mildly, but it didn’t bother me much
94. moderately-it wasn’t pleasant, but I can tolerate.
95. severely-I can barely tolerate it.
96. face flushed
97. not at all
98. mildly, but it didn’t bother me much
99. moderately-it wasn’t pleasant, but I can tolerate.
100. severely-I can barely tolerate it.
101. hot/cold sweats
102. not at all
103. mildly, but it didn’t bother me much
104. moderately-it wasn’t pleasant, but I can tolerate.
105. severely-I can barely tolerate it.

**This is the end of the survey, thank you for your participation.**
