# Supplementary material for: Analysis of anxiety-related factors amongst frontline dental staff during the COVID-19 pandemic in Yichang, China
Source: BMC Oral Health. 2020 Nov 26;20:342. doi: 10.1186/s12903-020-01335-9 (PMC7689639; doi:10.1186/s12903-020-01335-9)
Supplement: Supplementary file 2 — Additional file 2. [file 12903_2020_1335_MOESM2_ESM.docx]

**宜昌新冠疫情期间一线口腔医护人员工作近况和焦虑状况调查问卷**

当前全国各地，恢复正常生活和工作的进程正在稳步推进。但同时，新冠病毒的潜在风险仍然存在。一直以来，牙科医生和护士都是交叉感染的高危人群。特别是在当前情况下，他们承受的压力更大。本次研究希望通过了解一线牙科医护人员的工作现状和焦虑情况，为医院完善一线牙科医护人员的防护措施以及提供心理支持提供有效的参考建议。

本次问卷中包含一些个人信息和焦虑状态的问题，如有不适可以随时终止问卷。您的数据会被匿名搜集并且存在有密码保护的电脑上。

如有任何问题，可以联系：

- 刘蓓蓓，邮件：lpxblnottingham@qq.com
- 赵苏立，邮件：zhaosuli-9@163.com
- 孙榕灿，邮件：rongcan.sun@yale.edu

您需要是一位宜昌市一线牙科医护人员才能填写本问卷。

知晓以上信息后，您是否愿意参加这项调查？

1. 是
2. 否

**感谢您利用您的宝贵时间完成这个问卷，请您按照指示语进行填写，谢谢。**

1. 性别 [单选题]

1. 男
2. 女

2. 年龄 [填空题]

3. 岗位[填空题]

**请跟据您的工作情况填写以下问题，谢谢。**

4. 最近每周工作多少天 [填空题]

5. 最近我每天工作的时间是(小时) [填空题]

6. 最近我一般连续工作的时间是 (休息时间不少于5分钟为一次休息，连续工作时间请填小时) [填空题]

7. 我经常从事可产生大量气溶胶的操作[单选题]

1. 是
2. 否

8. 最近半年我与同事或者患者发生过激烈冲突 [单选题]

1. 是
2. 否

9. 我给疑似或确诊新冠的病人治疗过 [单选题]

1. 是
2. 否

10. 最近半年我的皮肤或者创面直接接触过患者的血液、体液、分泌物等污染物 [单选题]

1. 是
2. 否

11. 我现在工作的地方严格实施三级防护措施 [单选题]

1. 是
2. 否

**请根据您对新冠病毒的了解填写以下问题, 谢谢。**

12. 新冠感染主要症状 [单选题] （答案A）

1. 发热，干咳，乏力
2. 鼻塞，咽痛，乏力
3. 发热，咽痛，腹泻
4. 发热，鼻塞，咽痛

13. 新冠病毒潜伏期 [单选题] （答案B）

1. 3-7天
2. 1-14天
3. 1-28天
4. 大于28天

14. 新冠病毒的主要传播途径 [单选题] （答案A）

1. 飞沫，接触，密闭环境高浓度气溶胶
2. 飞沫，接触，粪便
3. 接触，密闭环境高浓度气溶胶，粪便
4. 飞沫，接触，密闭环境高浓度气溶胶，粪便

15. 下面哪个不是新冠病毒灭活途径 [单选题] （答案D）

1. 乙醚，75%乙醇，含氯消毒剂
2. 75%乙醇，含氯消毒剂，56摄氏度30分钟
3. 75%乙醇，含氯消毒剂，过氧乙酸
4. 75%乙醇，过氧乙酸，氯己定

16. 新冠病人出院标准 [单选题] （答案C）

1. 体温恢复正常3天以上、呼吸道症状明显好转
2. 临床症状缓解，体温正常，两次核酸检测都呈阴性
3. 体温恢复正常3天以上、呼吸道症状明显好转，肺部影像学显示炎症明显吸收, 连续两次呼吸道病原核酸检测阴性(采样时间间隔至少1天)
4. 体温恢复正常7天以上、呼吸道症状明显好转，肺部影像学显示炎症明显吸收, 连续两次呼吸道病原核酸检测阴性(采样时间间隔至少1天)

17. 易感染人群 [单选题] （答案D）

1. 儿童和老人
2. 老人
3. 青壮年和老人
4. 普遍易感染

**下面是关于焦虑一般症状的问题，请您仔细阅读下列各项，指出最近一周内（包括当天），被各种症状烦扰的程度，并选择相应的选项。**

18. 麻木或刺痛 [单选题]

1. 无
2. 轻度，无多大烦扰
3. 中度，感到不适但尚能忍受
4. 重度，只能勉强忍受

19. 感到发热 [单选题]

1. 无
2. 轻度，无多大烦扰
3. 中度，感到不适但尚能忍受
4. 重度，只能勉强忍受

20. 腿部颤抖 [单选题]

1. 无
2. 轻度，无多大烦扰
3. 中度，感到不适但尚能忍受
4. 重度，只能勉强忍受

21. 不能放松 [单选题]

1. 无
2. 轻度，无多大烦扰
3. 中度，感到不适但尚能忍受
4. 重度，只能勉强忍受

22. 害怕发生不好的事情 [单选题]

1. 无
2. 轻度，无多大烦扰
3. 中度，感到不适但尚能忍受
4. 重度，只能勉强忍受

23. 头晕 [单选题]

1. 无
2. 轻度，无多大烦扰
3. 中度，感到不适但尚能忍受
4. 重度，只能勉强忍受

24. 心悸或心率加快 [单选题]

1. 无
2. 轻度，无多大烦扰
3. 中度，感到不适但尚能忍受
4. 重度，只能勉强忍受

25. 心神不定 [单选题]

1. 无
2. 轻度，无多大烦扰
3. 中度，感到不适但尚能忍受
4. 重度，只能勉强忍受

26. 惊吓 [单选题]

1. 无
2. 轻度，无多大烦扰
3. 中度，感到不适但尚能忍受
4. 重度，只能勉强忍受

27. 紧张 [单选题]

1. 无
2. 轻度，无多大烦扰
3. 中度，感到不适但尚能忍受
4. 重度，只能勉强忍受

28. 窒息感 [单选题]

1. 无
2. 轻度，无多大烦扰
3. 中度，感到不适但尚能忍受
4. 重度，只能勉强忍受

29. 手发抖 [单选题]

1. 无
2. 轻度，无多大烦扰
3. 中度，感到不适但尚能忍受
4. 重度，只能勉强忍受

30. 摇晃 [单选题]

1. 无
2. 轻度，无多大烦扰
3. 中度，感到不适但尚能忍受
4. 重度，只能勉强忍受

31. 害怕失控 [单选题]

1. 无
2. 轻度，无多大烦扰
3. 中度，感到不适但尚能忍受
4. 重度，只能勉强忍受

32. 呼吸困难 [单选题]

1. 无
2. 轻度，无多大烦扰
3. 中度，感到不适但尚能忍受
4. 重度，只能勉强忍受

33. 害怕快要死去 [单选题]

1. 无
2. 轻度，无多大烦扰
3. 中度，感到不适但尚能忍受
4. 重度，只能勉强忍受

34. 恐慌 [单选题]

1. 无
2. 轻度，无多大烦扰
3. 中度，感到不适但尚能忍受
4. 重度，只能勉强忍受

35. 消化不良或腹部不适 [单选题]

1. 无
2. 轻度，无多大烦扰
3. 中度，感到不适但尚能忍受
4. 重度，只能勉强忍受

36. 昏厥 [单选题]

1. 无
2. 轻度，无多大烦扰
3. 中度，感到不适但尚能忍受
4. 重度，只能勉强忍受

37. 脸发红 [单选题]

1. 无
2. 轻度，无多大烦扰
3. 中度，感到不适但尚能忍受
4. 重度，只能勉强忍受

38. 出汗（不是因为暑热） [单选题]

1. 无
2. 轻度，无多大烦扰
3. 中度，感到不适但尚能忍受
4. 重度，只能勉强忍受

**问卷结束，谢谢参与！**
