# Supplementary material for: Analysis of anxiety-related factors amongst frontline dental staff during the COVID-19 pandemic in Yichang, China
Source: BMC Oral Health. 2020 Nov 26;20:342. doi: 10.1186/s12903-020-01335-9 (PMC7689639; doi:10.1186/s12903-020-01335-9)
Supplement: Supplementary file 3 — Additional file 3. [file 12903_2020_1335_MOESM3_ESM.docx]

**The questionnaire of the anxiety state among the general public in Yichang during the COVID-19 pandemic**

Recently, the daily life of the general public in Yichang is gradually resumed to normal condition. However, people still need to protect themselves to stay away from COVID-19, which may affect both their physical and mental health. Therefore, in our study, we would like to look at the anxiety state of the general public.

This questionnaire contains some questions asking about your personal information and your anxiety state, if you have any uncomfortable feeling, you can stop the survey at any time you like. Your data will be collected anonymously and will be stored on password-protected computers.

If you have any questions, please feel free to contact:

- Beibei Liu, Email: lpxblnottingham@qq.com
- Suli Zhao, Emial: zhaosuli-9@163.com
- Rongcan Sun, Email: rongcan.sun@yale.edu

To participate in this survey, you need to be one of the general public in Yichang.

After knowing the information above, are you willing to take part in this survey?

1. Yes
2. No

**Thank you for your precious time, please follow the instructions to complete the questions, thank you.**

1. Gender
2. Male
3. Female
4. Age (completion)
5. Occupation (completion)

**Below is a list of common symptoms of anxiety. Please carefully read each item in the list. Indicate how much you have been bothered by that symptom during the last 7 days, including today. Choose the most suitable description.**

1. numbness or tingling
2. not at all
3. mildly, but it didn’t bother me much
4. moderately-it wasn’t pleasant, but I can tolerate.
5. severely-I can barely tolerate it.
6. feeling hot
7. not at all
8. mildly, but it didn’t bother me much
9. moderately-it wasn’t pleasant, but I can tolerate.
10. severely-I can barely tolerate it.
11. wobbliness in legs
12. not at all
13. mildly, but it didn’t bother me much
14. moderately-it wasn’t pleasant, but I can tolerate.
15. severely-I can barely tolerate it.
16. unable to relax
17. not at all
18. mildly, but it didn’t bother me much
19. moderately-it wasn’t pleasant, but I can tolerate.
20. severely-I can barely tolerate it.
21. fear of worst happening
22. not at all
23. mildly, but it didn’t bother me much
24. moderately-it wasn’t pleasant, but I can tolerate.
25. severely-I can barely tolerate it.
26. dizzy or lightheaded
27. not at all
28. mildly, but it didn’t bother me much
29. moderately-it wasn’t pleasant, but I can tolerate.
30. severely-I can barely tolerate it.
31. heart pounding/racing
32. not at all
33. mildly, but it didn’t bother me much
34. moderately-it wasn’t pleasant, but I can tolerate.
35. severely-I can barely tolerate it.
36. unsteady
37. not at all
38. mildly, but it didn’t bother me much
39. moderately-it wasn’t pleasant, but I can tolerate.
40. severely-I can barely tolerate it.
41. terrified or afraid
42. not at all
43. mildly, but it didn’t bother me much
44. moderately-it wasn’t pleasant, but I can tolerate.
45. severely-I can barely tolerate it.
46. nervous
47. not at all
48. mildly, but it didn’t bother me much
49. moderately-it wasn’t pleasant, but I can tolerate.
50. severely-I can barely tolerate it.
51. feeling of chocking
52. not at all
53. mildly, but it didn’t bother me much
54. moderately-it wasn’t pleasant, but I can tolerate.
55. severely-I can barely tolerate it.
56. hands trembling
57. not at all
58. mildly, but it didn’t bother me much
59. moderately-it wasn’t pleasant, but I can tolerate.
60. severely-I can barely tolerate it.
61. shaking/unsteady
62. not at all
63. mildly, but it didn’t bother me much
64. moderately-it wasn’t pleasant, but I can tolerate.
65. severely-I can barely tolerate it.
66. fear of losing control
67. not at all
68. mildly, but it didn’t bother me much
69. moderately-it wasn’t pleasant, but I can tolerate.
70. severely-I can barely tolerate it.
71. difficulty in breathing
72. not at all
73. mildly, but it didn’t bother me much
74. moderately-it wasn’t pleasant, but I can tolerate.
75. severely-I can barely tolerate it.
76. fear of losing control
77. not at all
78. mildly, but it didn’t bother me much
79. moderately-it wasn’t pleasant, but I can tolerate.
80. severely-I can barely tolerate it.
81. difficulty in breathing
82. not at all
83. mildly, but it didn’t bother me much
84. moderately-it wasn’t pleasant, but I can tolerate.
85. severely-I can barely tolerate it.
86. fear of dying
87. not at all
88. mildly, but it didn’t bother me much
89. moderately-it wasn’t pleasant, but I can tolerate.
90. severely-I can barely tolerate it.
91. scared
92. not at all
93. mildly, but it didn’t bother me much
94. moderately-it wasn’t pleasant, but I can tolerate.
95. severely-I can barely tolerate it.
96. indigestion
97. not at all
98. mildly, but it didn’t bother me much
99. moderately-it wasn’t pleasant, but I can tolerate.
100. severely-I can barely tolerate it.
101. fainted/lightheaded
102. not at all
103. mildly, but it didn’t bother me much
104. moderately-it wasn’t pleasant, but I can tolerate.
105. severely-I can barely tolerate it.
106. hot/cold sweats
107. not at all
108. mildly, but it didn’t bother me much
109. moderately-it wasn’t pleasant, but I can tolerate.
110. severely-I can barely tolerate it.

**That is the end of the survey, thank you for your participation.**
