# Supplementary material for: Analysis of anxiety-related factors amongst frontline dental staff during the COVID-19 pandemic in Yichang, China
Source: BMC Oral Health. 2020 Nov 26;20:342. doi: 10.1186/s12903-020-01335-9 (PMC7689639; doi:10.1186/s12903-020-01335-9)
Supplement: Supplementary file 4 — Additional file 4. [file 12903_2020_1335_MOESM4_ESM.docx]

**宜昌地区新冠期间普通人群的焦虑状况调查问卷**

现在新冠防疫成为常态，宜昌地区普通群众积极参与防疫抗疫工作，人们的心理状况难免会受到影响。本调查关注宜昌普通人群的焦虑状况，希望通过调查结果反映普通人群的心理状态。

本次问卷中包含一些个人信息和焦虑状态的问题，如有不适可以随时终止问卷。您的数据会被匿名搜集并且存在有密码保护的电脑上。

如有任何问题，可以联系：

- 刘蓓蓓，邮件：lpxblnottingham@qq.com
- 赵苏立，邮件：zhaosuli-9@163.com
- 孙榕灿，邮件：rongcan.sun@yale.edu

您需要是一位普通宜昌群众才能参加本次调查。

知晓以上信息后，您是否愿意参加这项调查？

1. 是
2. 否

**感谢您利用您的宝贵时间完成这个问卷，请您按照指示语进行填写，谢谢。**

1. 性别 [单选题]
2. 男
3. 女
4. 年龄 [填空题]
5. 岗位[填空题]

**下面是关于焦虑一般症状的问题，请您仔细阅读下列各项，指出最近一周内（包括当天），被各种症状烦扰的程度，并选择相应的选项**^1^**。**

1. 麻木或刺痛 [单选题]
2. 无
3. 轻度，无多大烦扰
4. 中度，感到不适但尚能忍受
5. 重度，只能勉强忍受
6. 感到发热 [单选题]
7. 无
8. 轻度，无多大烦扰
9. 中度，感到不适但尚能忍受
10. 重度，只能勉强忍受
11. 腿部颤抖 [单选题]
12. 无
13. 轻度，无多大烦扰
14. 中度，感到不适但尚能忍受
15. 重度，只能勉强忍受
16. 不能放松 [单选题]
17. 无
18. 轻度，无多大烦扰
19. 中度，感到不适但尚能忍受
20. 重度，只能勉强忍受
21. 害怕发生不好的事情 [单选题]
22. 无
23. 轻度，无多大烦扰
24. 中度，感到不适但尚能忍受
25. 重度，只能勉强忍受
26. 头晕 [单选题]
27. 无
28. 轻度，无多大烦扰
29. 中度，感到不适但尚能忍受
30. 重度，只能勉强忍受
31. 心悸或心率加快 [单选题]
32. 无
33. 轻度，无多大烦扰
34. 中度，感到不适但尚能忍受
35. 重度，只能勉强忍受
36. 心神不定 [单选题]
37. 无
38. 轻度，无多大烦扰
39. 中度，感到不适但尚能忍受
40. 重度，只能勉强忍受
41. 惊吓 [单选题]
42. 无
43. 轻度，无多大烦扰
44. 中度，感到不适但尚能忍受
45. 重度，只能勉强忍受
46. 紧张 [单选题]
47. 无
48. 轻度，无多大烦扰
49. 中度，感到不适但尚能忍受
50. 重度，只能勉强忍受
51. 窒息感 [单选题]
52. 无
53. 轻度，无多大烦扰
54. 中度，感到不适但尚能忍受
55. 重度，只能勉强忍受
56. 手发抖 [单选题]
57. 无
58. 轻度，无多大烦扰
59. 中度，感到不适但尚能忍受
60. 重度，只能勉强忍受
61. 摇晃 [单选题]
62. 无
63. 轻度，无多大烦扰
64. 中度，感到不适但尚能忍受
65. 重度，只能勉强忍受
66. 害怕失控 [单选题]
67. 无
68. 轻度，无多大烦扰
69. 中度，感到不适但尚能忍受
70. 重度，只能勉强忍受
71. 呼吸困难 [单选题]
72. 无
73. 轻度，无多大烦扰
74. 中度，感到不适但尚能忍受
75. 重度，只能勉强忍受
76. 害怕快要死去 [单选题]
77. 无
78. 轻度，无多大烦扰
79. 中度，感到不适但尚能忍受
80. 重度，只能勉强忍受
81. 恐慌 [单选题]
82. 无
83. 轻度，无多大烦扰
84. 中度，感到不适但尚能忍受
85. 重度，只能勉强忍受
86. 消化不良或腹部不适 [单选题]
87. 无
88. 轻度，无多大烦扰
89. 中度，感到不适但尚能忍受
90. 重度，只能勉强忍受
91. 昏厥 [单选题]
92. 无
93. 轻度，无多大烦扰
94. 中度，感到不适但尚能忍受
95. 重度，只能勉强忍受
96. 脸发红 [单选题]
97. 无
98. 轻度，无多大烦扰
99. 中度，感到不适但尚能忍受
100. 重度，只能勉强忍受
101. 出汗（不是因为暑热） [单选题]
102. 无
103. 轻度，无多大烦扰
104. 中度，感到不适但尚能忍受
105. 重度，只能勉强忍受

**问卷结束，谢谢参与！**
